# The changing epidemiology of shigellosis in Australia, 2001–2019

**DOI:** 10.1371/journal.pntd.0010450

**Published:** 2023-03-01

**Authors:** Aaliya F. Ibrahim, Kathryn Glass, Deborah A. Williamson, Benjamin G. Polkinghorne, Danielle J. Ingle, Rose Wright, Martyn D. Kirk

**Affiliations:** 1 National Centre for Epidemiology and Population Health, Australian National University, Canberra, Australia; 2 Office of Health Protection, Australian Government Department of Health and Aged Care, Canberra, Australia; 3 Victorian Infectious Diseases Reference Laboratory, Royal Melbourne Hospital at The Peter Doherty Institute for Infection and Immunity, Melbourne, Australia, Melbourne, Australia; 4 Department of Infectious Diseases, The University of Melbourne at The Peter Doherty Institute for Infection and Immunity, Melbourne, Australia; 5 Department of Microbiology and Immunology, The University of Melbourne at The Peter Doherty Institute for Infection and Immunity, Melbourne, Australia; Tel Aviv University Sackler Faculty of Medicine, ISRAEL

## Abstract

Shigellosis is an increasing cause of gastroenteritis in Australia, with prolonged outbreaks reported in remote Aboriginal and Torres Strait Islander (hereafter “First Nations”) communities and among men who have sex with men (MSM) in major cities. To determine associations between *Shigella* species and demographic and geographic factors, we used multivariate negative binomial regression to analyse national case notifications of shigellosis from 2001 to 2019.

Between 2001 and 2019, Australian states and territories reported 18,363 shigellosis cases to the National Notifiable Diseases Surveillance System (NNDSS), of which age, sex and organism information were available for >99% (18,327/18,363) of cases. Of the cases included in our analysis, 42% (7,649/18,327) were *S*. *sonnei*, 29% (5,267/18,327) were *S*. *flexneri*, 1% (214/18,327) were *S*. *boydii*, less than 1% (87/18,327) were *S*. *dysenteriae*, and species information was unknown for 28% (5,110/18,327) of cases. Males accounted for 54% (9,843/18,327) of cases, and the highest proportion of cases were in children aged 0–4 years (19%; 3,562/18,327). Crude annual notification rates ranged from 2.2 cases per 100,000 in 2003 and 2011 to 12.4 cases per 100,000 in 2019. Nationally, notification rates increased from 2001 to 2019 with yearly notification rate ratios of 1.04 (95% CI 1.02–1.07) for *S*. *boydii* and 1.05 (95% CI 1.04–1.06) for *S*. *sonnei*. Children aged 0–4 years had the highest burden of infection for *S*. *flexneri*, *S*. *sonnei* and *S*. *boydii*; and males had a higher notification rate for *S*. *sonnei* (notification rate ratio 1.24, 95% CI 1.15–1.33). First Nations Australians were disproportionately affected by shigellosis, with the notification rate in this population peaking in 2018 at 92.1 cases per 100,000 population. Over the study period, we also observed a shift in the testing method used to diagnose shigellosis, with culture independent diagnostic testing (CIDT) increasing from 2014; this also coincided with an increase in notifications of untyped *Shigella*. This change in testing methodology may have contributed to the observed increase in shigellosis notifications since 2014, with CIDT being more sensitive than culture dependent testing methods.

The findings of this study provide important insights into the epidemiological characteristics of shigellosis in Australia, including identification of high-risk groups. This can be used to inform public health prevention and control strategies, such as targeted communication programs in First Nations communities and places with high levels of interaction between young children, such as childcare centres. Our study findings also highlight the implications of culture independent testing on shigellosis surveillance, particularly a reduction in the availability of species level information. This emphasises the continued importance of culture dependant testing for national surveillance of shigellosis.

## Author summary

*Shigella* spp. causes gastroenteritis and is responsible for significant morbidity and mortality worldwide. In Australia, largescale shigellosis outbreaks have been reported in First Nations communities and among men who have sex with men in recent years. We undertook an analysis of the trends in shigellosis cases in Australia over a 19-year period to understand the epidemiology of the disease, including which population groups were primarily impacted, and when and where cases were occurring. We also analysed trends in the diagnostic methods used to detect shigellosis over time, to determine whether changing detection methods had any impact on the surveillance of shigellosis cases at the national level. We found that notification rates of two species of *Shigella*, i.e. *S*. *boydii* and *S*. *sonnei*, significantly increased over the study period; the highest notification rate of shigellosis was in young children; and that First Nations Australians were disproportionately impacted by shigellosis. We also observed a shift in the testing methods used to diagnose shigellosis, with culture independent methods increasing from 2014. Our findings will assist in targeting prevention and control interventions to the most impacted groups and highlight the continued importance of culture dependant testing for national surveillance of shigellosis.

## Introduction

Shigellosis is caused by gram-negative *Shigella* spp. bacteria, which is primarily transmitted through the faecal-oral route by direct or indirect contact with faecal matter [1]. There are four species of *Shigella* bacteria, characterised by their O antigen type and biochemical properties: *S*. *dysenteriae*, *S*. *flexneri*, *S*. *boydii* and *S*. *sonnei* [2]. Shigellosis outbreaks have been associated with consumption of contaminated food and water; contact with an infected person, particularly in settings with poor hygiene, through sexual contact, or in substandard and crowded living conditions; and recreational contact with contaminated water supplies [3,4]. The spread of *Shigella* can also occur via flies, which act as a mechanical vector for the organism, however this mode of transmission has not been widely reported in Australia [5,6]. *Shigella* have a low infectious dose between 10 to 100 organisms, and the clinical presentations of infection can vary from mild watery diarrhoea to severe dysentery (bloody diarrhoea) compounded by systemic complications such as electrolyte imbalance, seizures, fever, nausea and haemolytic uraemic syndrome [7]. While shigellosis is often a self-limiting disease, treatment with antibiotics may shorten the duration of the illness and prevent prolonged bacterial shedding, and is recommended for those with severe disease or who are immunocompromised [8,9]. As per Australian guidelines, treatment is also recommended to reduce transmission in key risk groups, including children under the age of six years, healthcare workers, and people living or working in residential aged-care facilities, prisons, and other residential facilities [10]. Globally, *Shigella* species are a leading cause of diarrhoeal mortality, causing an estimated 200,000 deaths in 2016 [11]. The greatest burden of shigellosis is in young children in low- and middle-income countries, where access to good nutrition, clean water, adequate sanitation and healthcare is limited [12]. In contrast, the highest rates of shigellosis in high-income countries typically occur through person-to-person contact in crowded communities and childcare centres, in travellers returning from overseas or in MSM [9,13]. Increasing transmission of *Shigella* among MSM, particularly antimicrobial resistant strains, have been reported in several high-income countries, including the United States, Canada, Germany and the United Kingdom [14–17]. The most commonly reported species in high-income countries is *S*. *sonnei*, with this species accounting for over 80% of shigellosis infections in the United States and Israel, and over 50% of infections in the United Kingdom [18–20]. Australian national surveillance for shigellosis show a gradual increase in shigellosis case numbers until 2019, when it more than doubled compared to the 5-year rolling mean [21]. Outbreaks have also been reported in First Nations communities in central Australia and among MSM [22,23].

Stool culture resulting in the isolation of *Shigella* remains the gold-standard method for obtaining a definitive laboratory diagnosis of shigellosis. However, since 2013 there has been an increasing uptake of culture independent diagnostic testing (CIDT) methods such as multiplex polymerase chain reaction (PCR) tests [24]. This change in testing practices has implications for public health surveillance of a wide range of enteric pathogens, including *Shigella*. Currently, PCR assays for detecting *Shigella* target the invasion plasmid antigen H (*ipaH)* gene, which is also common to entero-invasive *Escherichia coli* (EIEC) [25]. This means that such tests are unable to differentiate between these two organisms, which are closely related and genetically constitute the same species, but typically manifest different symptoms, with EIEC being milder [26]. Furthermore, *Shigella* can only be identified to the species and biotype level if an isolate is cultured. Therefore, while PCR testing to detect *Shigella* is more sensitive than traditional bacterial culture methods, the ability to obtain more detailed information, such as antimicrobial susceptibility and epidemiological typing, is compromised. The increasing uptake of CIDT is likely to have implications on the number, accuracy and completeness (with respect to species/biotype data) of surveillance data for shigellosis.

Despite *Shigella* being an increasing cause of gastroenteritis in Australia, there is very limited published research on the epidemiology of *Shigella* at a national level over a longer time frame. In this study, we describe trends in notifications of shigellosis from 2001 to 2019 to understand demographic and geographic factors, including sex, age group, Indigenous status and Australian states and territories. Additionally, we evaluate the impact of CIDT on national surveillance of shigellosis.

## Methods

### Ethics statement

We obtained ethical approval for this study from the Australian National University Human Research Ethics Committee [protocol 2018/560] and the ACT Health Human Research Ethics Committee’s Low Risk Sub-Committee [2018/ETH/00158]. No participants were recruited for this study and all data used was previously collected during public health investigations. Given that de-identified data was collected, representing infections occurring many years ago, it was not be feasible to seek individual consent, nor to inform participants about the use of data in this study.

### Data sources

We analysed shigellosis case notifications made to the National Notifiable Diseases Surveillance System (NNDSS) from 2001 to 2019 by all Australian states and territories. Australia is comprised of eight jurisdictions: New South Wales (NSW), Queensland (QLD), South Australia (SA), Tasmania (TAS), Victoria (VIC) and Western Australia (WA); and two territories: the Australian Capital Territory (ACT) and Northern Territory (NT).

Shigellosis is a nationally notifiable disease in Australia, and states and territories report all confirmed and probable cases to the Australian Government Department of Health through the NNDSS. The national case definition requires that a confirmed case of shigellosis has *Shigella* species isolated; or that *Shigella* is detected by nucleic acid testing in combination with epidemiological evidence [27]. The addition of ‘probable’ cases in the surveillance case definition, i.e. detection of *Shigella* by nucleic acid testing only, was implemented from 1 July 2018; prior to this, only confirmed cases were required to be notified. In this study, we did not include ‘probable’ cases. However, it should be noted, prior to the implementation of the new case definition in July 2018, testing and notification practices varied by jurisdiction. The old shigellosis case definition defined laboratory definitive evidence as ‘*isolation or detection of Shigella species*’. Based on this, most states interpreted a PCR positive result as not a detection and waited for a positive result from reflex culture before making a notification. However, a few states sent all these case notifications to the NNDSS marked as ‘probable’, which were recorded by the NNDSS system as a ‘confirmed’ case. This primarily occurred from late 2017 until the implementation of the new case definition in mid- 2018. As not all these ‘probable’ cases can be identified in the NNDSS dataset, they were not excluded from this study. In addition to differences in the application of the case definition, the testing methodologies used to detect *Shigella* also varied by jurisdiction and were implemented inconsistently between states and territories over time.

New South Wales–Australia’s most populous state–was the last Australian jurisdiction to make shigellosis reportable, which occurred in 2001. Therefore, we included de-identified data on all confirmed shigellosis case notifications reported to the NNDSS from 2001 to 2019 with the following variables: state; sex; 5 year age-group; month and year of diagnosis; country of acquisition; organism; biotype/subtype; Indigenous status and laboratory diagnosis method. NNDSS data were provided by the Office of Health Protection, Department of Health, on behalf of the Communicable Diseases Network Australia. The month and year of diagnosis was calculated from the diagnosis date: a derived field representing the onset date of the illness or the earliest of the specimen collection date, the date of report, or the date of receipt at the state or territory health authority. The data fields in the laboratory diagnosis method variable were consolidated into the following categories depending on whether culture and nucleic acid testing were reported: PCR; culture; PCR and culture; unknown (including blanks); and other (any fields with values that did not include culture, PCR or unknown).

### Analysis

The primary aim of the analysis was to examine the trend in notification rates of shigellosis over time. Cases where sex, age-group and organism were unknown were excluded from the analysis. We calculated rates of illness per 100,000 population using mid-year residential population estimates from the Australian Bureau of Statistics (ABS) for the years 2001 to 2019.

We used a multivariate negative binomial regression model to estimate notification rate ratios (NRR) by sex, age and state and territory, with significance defined as a p-value of less than 0.05. An interaction term was included in the model to analyse the trend over time for each state and territory, with diagnosis date included as a continuous variable. As NSW is the most populous Australian jurisdiction, we applied it as the reference group for the jurisdictional analysis in the model. Due to low (<5) or no cases of *S*. *dysenteriae* in ACT, NT and TAS, these states were removed from the regression model for *S*. *dysenteriae*. StataCorp Stata 15 and Microsoft Excel 2016 were used for analysis.

## Results

Between 2001 and 2019, Australian states and territories reported 18,363 shigellosis cases to the NNDSS, of which age, sex and organism information were available for >99% (18,327/18,363) of cases. Of the cases included in the analysis, 42% (7,649/18,327) were *S*. *sonnei*, 29% (5,267/18,327) were *S*. *flexneri*, 1% (214/18,327) were *S*. *boydii*, and less than 1% (87/18,327) were *S*. *dysenteriae* (Table 1). Species information was unknown for 28% (5,110/18,327) of cases, with 79% (4,024/5,110) of these unknown species occurring in the years 2016–2019.

**Fig 1:**
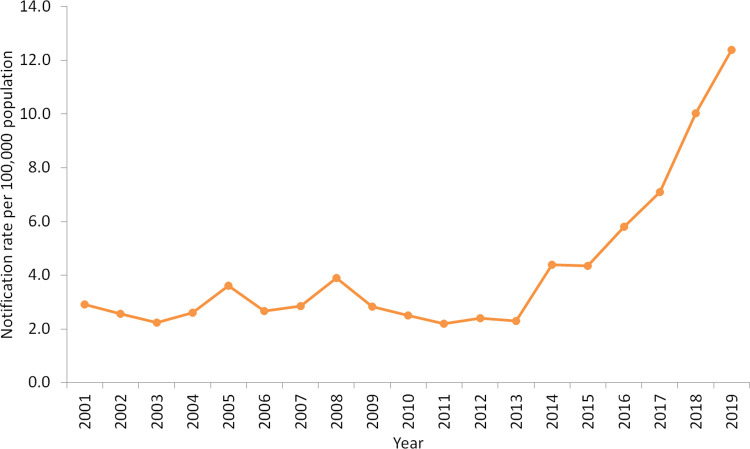
Crude notification rate of shigellosis per 100,000 population, Australia, 2001–2019.

**Fig 2:**
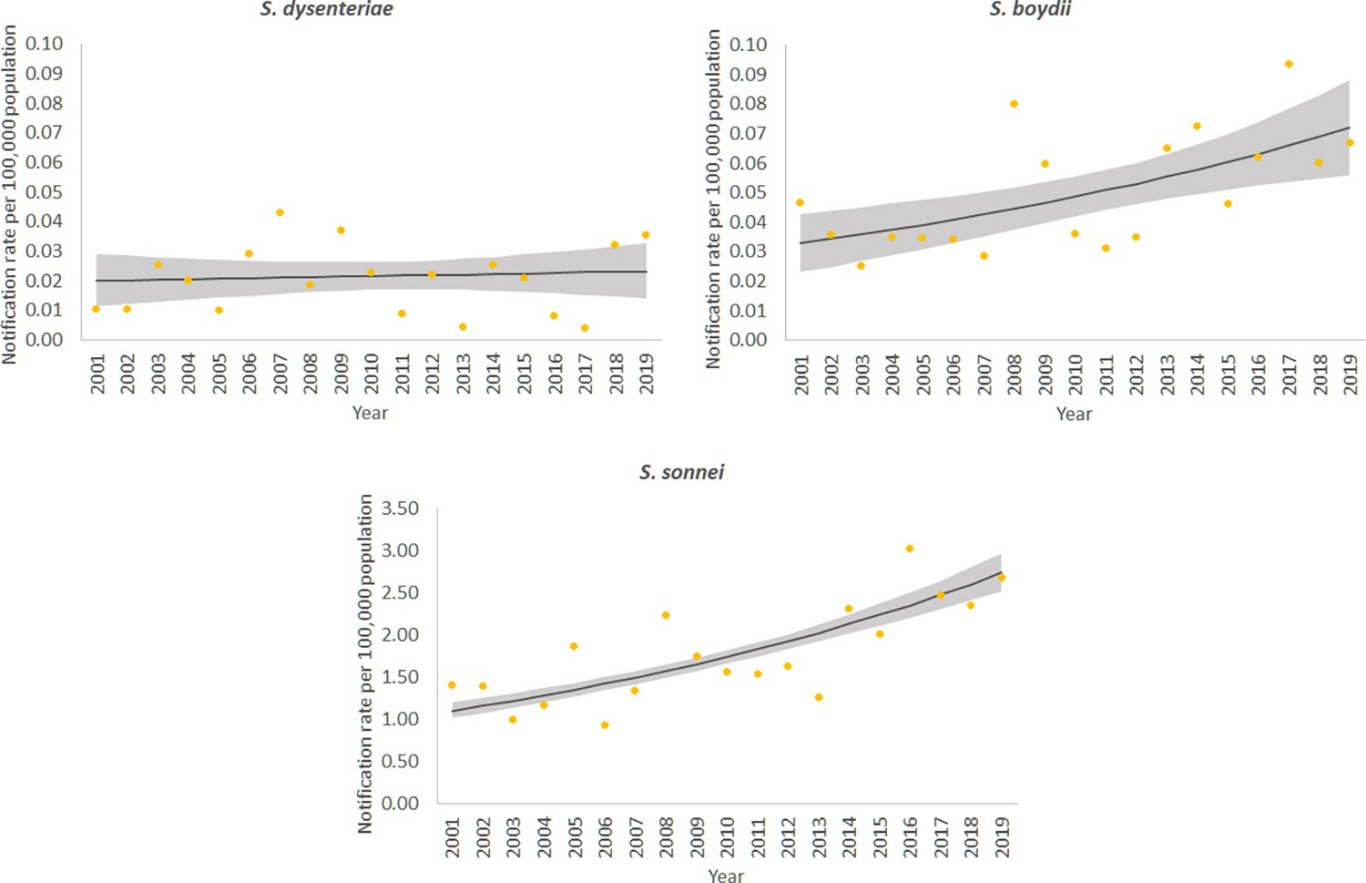
Crude notification rates (dots) and negative binomial regression margins plots (lines with 95% CI) of *S*. *boydii*, *S*. *sonnei* and *S*. *dysenteriae*, Australia 2001–2019. *(Note the differing scale of the y-axis for S. boydii and S. dysenteriae, compared to S. sonnei. Grey area represents 95% confidence intervals.)*.

Nationally, crude annual notification rates ranged from 2.2 cases per 100,000 in 2003 and 2011 to 12.4 cases per 100,000 in 2019 (Fig 1). Rates remained relatively stable between 2001 and 2013, while from 2013 to 2019 there was a greater than five-fold increase in the overall crude notification rate of shigellosis in Australia. The regression model for *S*. *flexneri* did not fit the data well, which is likely due to outbreaks occurring in the latter years of surveillance in specific states. The results of the regression model for *S*. *flexneri* are available in the supplementary material (S2 Fig and S1 Table). Nationally, notification rates increased from 2001 to 2019 for *S*. *boydii* and *S*. *sonnei* with yearly notification rate ratios of 1.04 (95% CI 1.02–1.07) and 1.05 (95% CI 1.04–1.06), respectively (Fig 2). The NRR for *S*. *dysenteriae* indicated there was an increase in notification rates between 2001 and 2019, although there was less precision around the interval estimate (NRR 1.01; 95% CI 0.97–1.05).

**Table 1:** Shigellosis notifications and population size by age group, sex and jurisdiction, Australia, 2001–2019. *(Note*: *Excludes cases for whom sex*, *age and organism were unknown*. *Population sizes are the ABS mid-year estimated residential populations for 2019)*.

	*S*. *flexneri*	*S*. *sonnei*	*S*. *dysenteriae*	*S*. *boydii*	*Shigella* untyped	Population size
**Age groups**						
0–4	1,349	1,250	6	36	921	1,568,012
5–9	449	491	4	12	307	1,618,281
10–14	183	207	4	5	163	1,555,558
15–19	199	215	7	8	182	1,498,871
20–24	369	502	9	10	418	1,749,579
25–29	414	812	8	22	495	1,906,651
30–34	407	814	9	17	421	1,892,645
35–39	377	694	8	16	365	1,782,081
40–44	316	601	6	16	299	1,595,917
45–49	296	546	5	17	274	1,679,382
50–54	255	506	8	13	277	1,535,463
55–59	208	382	4	13	296	1,547,756
60–64	164	238	3	15	228	1,391,597
65–69	114	207	3	7	202	1,227,169
70–74	78	96	0	3	132	1,057,980
75–79	46	49	1	1	72	734,222
80–84	26	19	1	0	30	505,017
85+	17	20	1	3	28	514,905
**Jurisdiction**						
ACT	27	77	0	1	60	426,285
NSW	717	1,925	28	77	1,008	8,087,379
NT	1,541	877	0	4	723	246,143
QLD	612	1,466	17	22	779	5,093,884
SA	471	461	5	19	399	1,752,681
TAS	29	40	1	1	57	534,575
VIC	783	1,759	21	67	1,743	6,596,880
WA	1,087	1,044	15	23	341	2,623,259
**Sex**						
Female	2,577	3,165	43	98	2,601	12,784,211
Male	2,690	4,484	44	116	2,509	12,576,875

**Fig 3:**
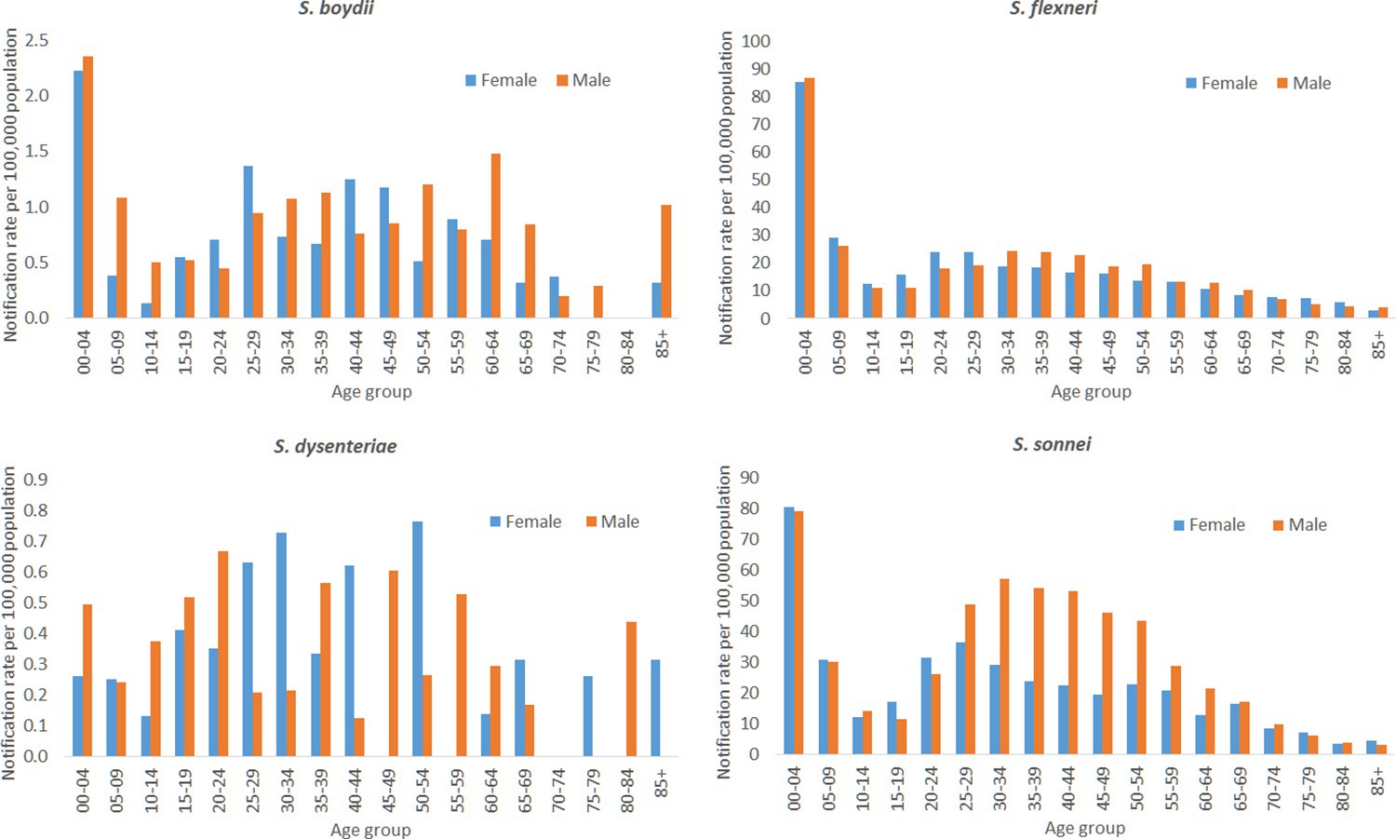
Crude notification rate of shigellosis per 100,000 population, by sex and age, Australia, 2001–2019.

Males accounted for 54% (9,843/18,327) of cases. Over the 19-year period, the overall crude notification rate of shigellosis was higher in males than females in the eastern states (ACT, NSW, QLD, and VIC); while the western states and territories experienced higher rates in females (S1 Fig). In the state and territory regression model, there was a significantly higher notification rate in males than females for *S*. *sonnei* (NRR 1.24; 95% CI 1.15–1.33); while there was no significant difference between the sexes for the other species (Table 2). For *S*. *sonnei*, the crude notification rate among males was notably higher than the rate in females for those aged 30 to 64 years, while in all other age groups the rate was similar for both sexes (Fig 3).

**Table 2:** Notification rate ratios estimated using negative binomial regression of *S*. *sonnei*, *S*. *dysenteriae* and *S*. *boydii* by gender, age, state and time, 2001–2019.

	*S*. *sonnei*	*S*. *dysenteriae*	*S*. *boydii*
	NRR (95% CI)	NRR (95% CI)	NRR (95% CI)
**Age groups (reference 0–4)**			
5–9	0.36 (0.30–0.42)	0.68 (0.19–2.43)	0.34 (0.17–0.65)
10–14	0.15 (0.12–0.18)	0.68 (0.19–2.43)	0.14 (0.06–0.36)
15–19	0.15 (0.12–0.18)	1.18 (0.39–3.55)	0.22 (0.10–0.48)
20–24	0.31 (0.26–0.37)	1.40 (0.49–3.98)	0.25 (0.13–0.51)
25–29	0.45 (0.38–0.53)	1.20 (0.41–3.50)	0.54 (0.32–0.91)
30–34	0.44 (0.37–0.52)	1.33 (0.47–3.80)	0.42 (0.23–0.74)
35–39	0.38 (0.32–0.45)	1.22 (0.42–3.58)	0.41 (0.23–0.73)
40–44	0.34 (0.29–0.41)	0.92 (0.29–2.90)	0.41 (0.23–0.74)
45–49	0.31 (0.26–0.37)	0.78 (0.24–2.61)	0.45 (0.25–0.79)
50–54	0.32 (0.27–0.38)	1.33 (0.45–3.88)	0.36 (0.19–0.68)
55–59	0.27 (0.23–0.33)	0.72 (0.20–2.60)	0.39 (0.21–0.74)
60–64	0.21 (0.17–0.26)	0.63 (0.16–2.55)	0.52 (0.28–0.95)
65–69	0.22 (0.18–0.27)	0.76 (0.19–3.07)	0.29 (0.13–0.65)
70–74	0.13 (0.10–0.16)	N/A	0.15 (0.05–0.50)
75–79	0.09 (0.06–0.12)	0.41 (0.05–3.42)	0.07 (0.01–0.49)
80–84	0.05 (0.03–0.08)	0.56 (0.07–4.73)	N/A
85+	0.05 (0.03–0.09)	0.62 (0.07–5.19)	0.30 (0.09–0.98)
**Jurisdiction (reference = NSW)**			
ACT	0.92 (0.53–1.58)	N/A	0.20 (0.00–34.21)
NT	25.31 (19.85–32.27)	N/A	2.40 (0.27–21.29)
QLD	0.85 (0.67–1.09)	1.25 (0.36–4.31)	0.42 (0.13–1.39)
SA	2.26 (1.73–2.96)	1.62 (0.30–8.89)	2.80 (1.03–7.62)
TAS	0.45 (0.23–0.88)	N/A	0.21 (0.00–20.35)
VIC	0.99 (0.78–1.24)	1.47 (0.47–4.56)	2.01 (0.96–4.21)
WA	2.87 (2.26–3.63)	0.96 (0.22–4.22)	0.97 (0.31–3.05)
**Trend over time by state and territory (2001–2019)**			
ACT	1.04 (1.00–1.09)	N/A	1.09 (0.74–1.62)
NSW	1.06 (1.05–1.08)	1.02 (0.95–1.09)	1.07 (1.03–1.12)
NT	0.98 (0.96–1.00)	N/A	1.03 (0.86–1.24)
QLD	1.10 (1.08–1.11)	1.00 (0.91–1.09)	1.08 (0.99–1.17)
SA	0.99 (0.97–1.01)	0.94 (0.80–1.11)	0.98 (0.90–1.06)
TAS	1.03 (0.97–1.09)	N/A	1.07 (0.73–1.55)
VIC	1.08 (1.06–1.10)	0.97 (0.90–1.06)	1.02 (0.97–1.06)
WA	1.01 (0.99–1.02)	1.07 (0.97–1.19)	1.07 (0.98–1.16)
**Sex (reference = female)**			
Male	1.24 (1.15–1.33)	1.03 (0.67–1.58)	1.18 (0.90–1.55)

Nationally, the highest proportion of shigellosis notifications occurred in children aged 0–4 years (19%; 3,562/18,327). By species, notification rates of *S*. *boydii*, *S*. *flexneri* and *S*. *sonnei* were highest in children aged 0–4 years (Fig 3). The highest rate of cases for *S*. *dysenteriae* occurred in those aged 20–24 years, however this was not significantly different from the 0–4 year age reference group (NRR 1.40; 95% CI 0.49–3.98). Comparing the two most prevalent species, in those aged 25–59 years, the notification rate of *S*. *sonnei* was significantly higher than *S*. *flexneri*, while there was no significant difference in the notification rate of these two species in the other age groups (S3 Fig).

**Fig 4:**
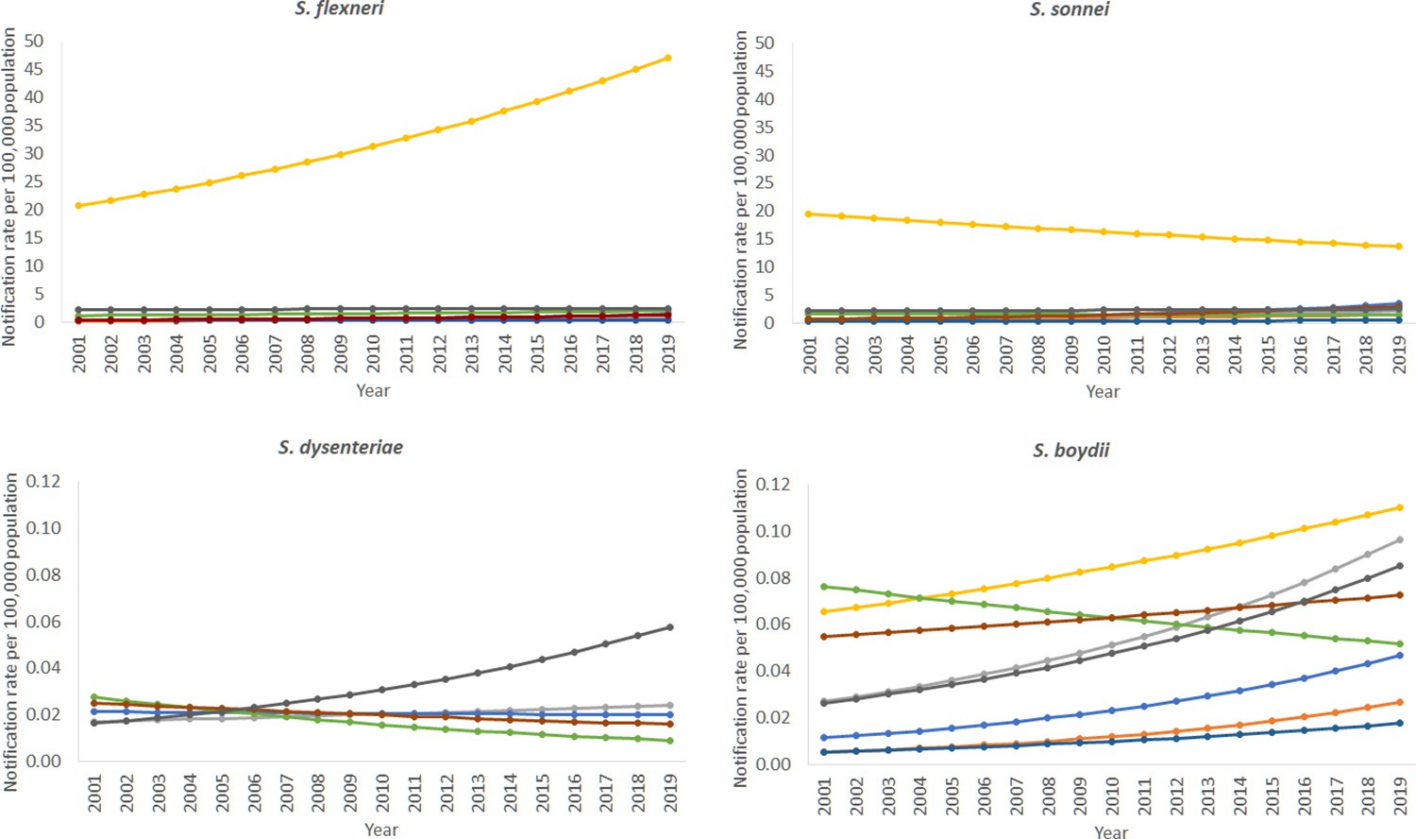
Negative binomial regression margins plot of *S*. *flexneri*, *S*. *sonnei*, *S*. *dysenteriae* and *S*. *boydii* predicted notification rates by state and territory, Australia 2001–2019.

Notification rates varied by jurisdiction, with the NT having considerably higher rates than other states and territories. In 2019, the rate of shigellosis in the NT was 120.3 cases per 100,000 population, nearly 10 times the overall national notification rate (12.4 cases per 100,000 population) (S4 Fig). After the NT, in 2019, notification rates were highest in WA (14.8 cases per 100,000 population), followed by QLD (12.0 cases per 100,000 population). At the start of the period, notification rates of *S*. *sonnei* were significantly higher in the states and territories in the west of Australia, that is NT (NRR 25.31; 95% CI 19.85–32.27), WA (NRR 2.87; 95% CI 2.26–3.63) and SA (NRR 2.26; 95% CI 1.73–2.96), compared to the south eastern continental states and territories. The notification rate of this species in TAS was significantly lower than the other jurisdictions (NRR 0.45; 95% CI 0.23–0.88). For *S*. *dysenteriae* and *S*. *boydii*, there was considerable uncertainty in the results due to low case numbers, making it difficult to conclude there was a difference in the notification rate between states and territories (Fig 4). The NT, SA and VIC all had higher rates of *S*. *boydii*, although there was considerable imprecision in the NRR.

Looking at trends over time, from 2001 to 2019, notification rates of *S*. *sonnei* significantly increased in NSW, QLD and VIC (Table 2). NSW also experienced a significant increase in *S*. *boydii*, with an NRR of 1.07 (95% CI 1.03–1.12), although results were imprecise. There was no significant change in the notification rates of *S*. *dysenteriae* for any state or territory over the 19 year period (Table 2). Regression lines plotted against the crude notification rates for individual states and territories are available in S5 Fig.

**Fig 5:**
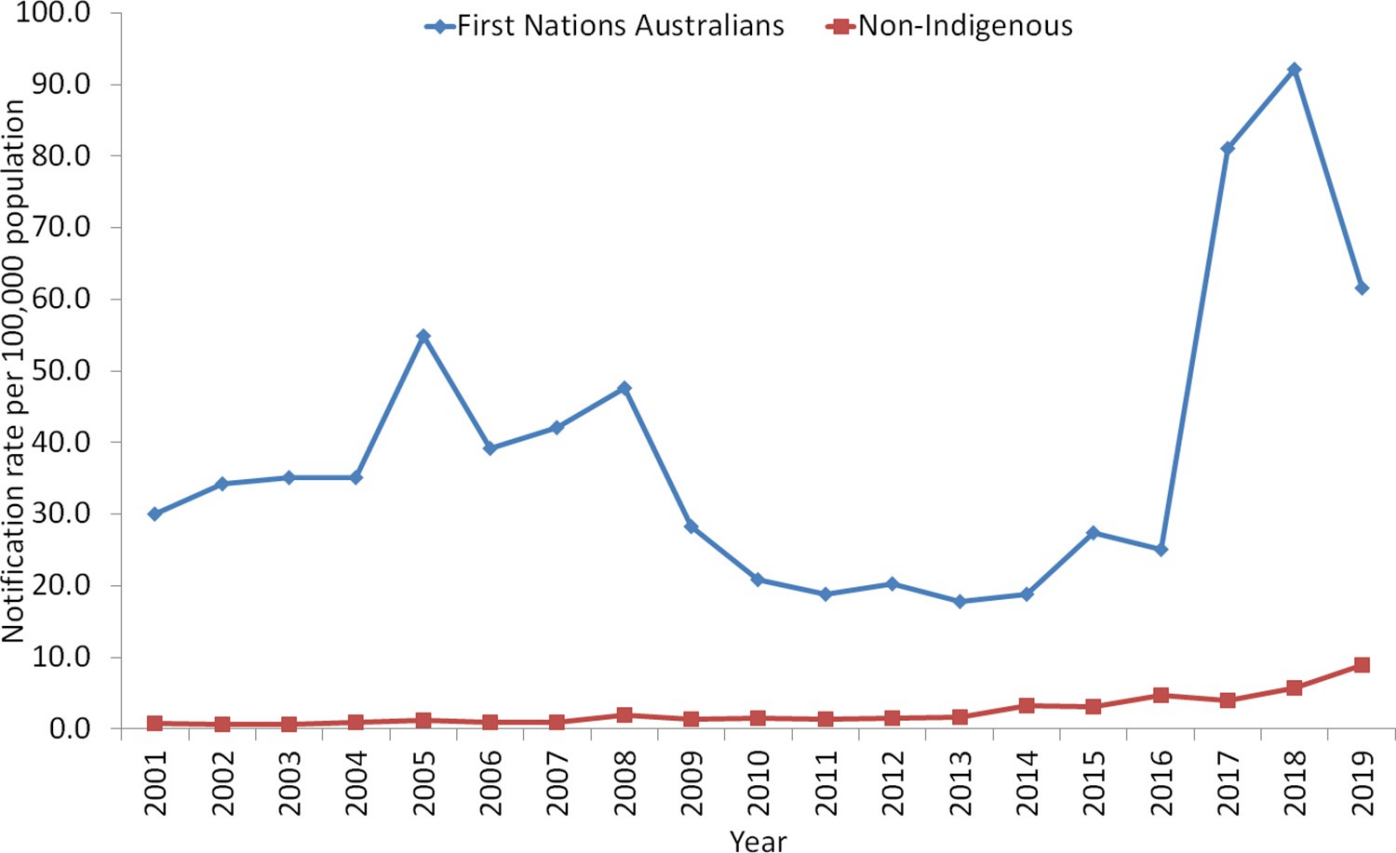
Notification rate of shigellosis per 100,000 population, by Indigenous status, Australia, 2001–2019.

Indigenous status was available for 85% (15,529/18,327) of cases included in the analysis. There were higher rates of shigellosis among First Nations Australians when compared to the non-Indigenous population (Fig 5). Between 2016 and 2018 there was a greater than 3-fold increase in the notification rate in First Nations Australians, peaking at 92.1 cases per 100,000 in 2018.

Information on laboratory diagnostic method was available for 87% of cases (16,004/18,327). Between 2001 and 2013, most nationally notified cases of shigellosis were reported to be diagnosed using culture only, peaking at 95% (786/829) of cases in 2008. From 2014 to 2019 there was a decrease in the proportion of cases diagnosed via culture methods, ranging from 54% (554/1,033) in 2014 to 27% (835/3,142) in 2019, due to increasing use of CIDT methods. This led to increased reporting of untyped *Shigella*, with >63-fold increase in cases of untyped *Shigella* from 2013 (n = 30) to 2019 (n = 1,902). At a jurisdictional level, all states and territories experienced a dramatic increase in untyped *Shigella* in the past few years (S6 Fig).

Country of acquisition data was available for 53% (9,782/18,327) of cases. Due to the poor completeness of this field, no further analysis was undertaken using this variable.

## Discussion

Notification rates of shigellosis have increased considerably in Australia over the past 19 years (2001–2019), particularly since the introduction of CIDT. The reported rate of all species increased over the time period, except for *S*. *dysenteriae*. The most common species in Australia was *S*. *sonnei* (42% all cases), followed by *S*. *flexneri* (29% of cases), consistent with the reported global burden of shigellosis [12]. While *S*. *flexneri* is traditionally linked to low and middle income countries and *S*. *sonnei* to high income countries, in recent years the expansion of *S*. *sonnei* has been documented in many economically transitional regions in Asia, Latin America and the Middle East [28]. Globally, data on the epidemiology of *S*. *boydii* is limited, with this species being predominantly endemic in the Indian Subcontinent [29]. Notification rates of *S*. *dysenteriae* in Australia were very low compared to *S*. *sonnei* and *S*. *flexneri*, with *S*. *dysenteriae* rarely being isolated in current surveillance globally [12]. *S*. *dysenteriae* serotype 1, which is the only *Shigella* species and serotype that expresses the Shiga toxin gene, has high epidemic potential and has been identified as a cause of several large scale outbreaks of dysentery, particularly in settings associated with civil unrest [30–34]. This strain is also notorious for developing drug resistance, with epidemic strains typically resistant to multiple antibiotics [35,36]. The epidemiology of *S*. *dysenteriae* is distinct from the other species of *Shigella*, with epidemics tending to disappear and then reappear years later, with few sporadic cases reported in between outbreaks [37,38]. While large scale outbreaks of *S*. *dysenteriae* serotype 1 were prevalent in the second half of the twentieth century in Asia, Africa and Central America, there has been a drastic decline in the incidence of *S*. *dysenteriae* in the last few decades, which remains largely unexplained [7,34,39].

The relative importance of *Shigella* species in Australia varied by jurisdiction, with western jurisdictions, particularly the Northern Territory (NT) having higher rates across all years. The NT is unique demographically, with this jurisdiction having the highest proportion of its residents living in remote and very remote areas (approximately 40%), compared to the other states and territories [40]. It is widely documented that people living in remote areas have poorer health outcomes, including higher rates of hospitalisation and death, and also have more limited access to, and use of, health care services [41,42]. In addition to having the highest proportion of remote residents, the NT also has the highest proportion of First Nations Australians among its population (an estimated 32%), with over 75% of First Nations Australians in the NT living in remote or very remote areas [43,44]. In Australia, notification rates of shigellosis were higher in First Nations Australians than the non-Indigenous population, which was consistent throughout the period of surveillance. The disparity between the notification rate in First Nations Australians and the non-Indigenous population was particularly pronounced between 2017–2019, likely due to an outbreak of *S*. *flexneri* 2b that occurred among First Nations communities in central Australia, predominantly in the NT [22]. Antimicrobial resistance has also been reported in endemic strains of *Shigella* affecting First Nations Australians [45]. An estimated 18% of First Nations Australians are reported to live in overcrowded households, with the rates of overcrowding highest among those living in remote (26%) and very remote areas (51%) [46]. Overcrowding and lack of safe removal and treatment of sewage has shown to be positively associated with gastrointestinal infections in First Nations Australian households [47]. A multifaceted and preventative approach is needed to address the disproportional incidence of infectious diseases, including shigellosis, in First Nations communities.

Consistent with global trends, the burden of shigellosis in Australia was highest in children aged less than five years [31,48]. Children in this age group are unable to practice good personal hygiene and are yet to have acquired immunity due to a lack of previous exposure [49,50]. As shigellosis is typically a self-limiting disease, reported cases in NNDSS are likely to underrepresent the true incidence of disease. In general, notification data represent only those cases for which health care was sought, a test conducted, and a diagnosis made, followed by a notification to health authorities. Therefore, the higher notification rate of shigellosis in children may also be due to children being overrepresented in the NNDSS dataset due to a higher chance of receiving medical care when unwell, compared to adults. Additionally, the low infectious dose of *Shigella* results in person-to-person transmission, which often occurs between children in childcare settings through close contact [7,51]. Transmission in these settings can also occur due to inadequate handwashing after nappy changing or defecation, or faecal contamination of play areas or nappy changing surfaces [49,51]. High incidence among children can also lead to increased transmission to their carers. Compared to the eastern jurisdictions, the western states and territories had higher rates of shigellosis among females. This potentially reflects an underlying difference in the epidemiology of shigellosis in these states due to higher rates in children, which in turn resulted in higher rates of infection among carers, who are more likely to be female.

The eastern states of Australia had higher notification rates of shigellosis in males than females. This is likely to reflect ongoing transmission among MSM in these states, with outbreaks occurring over several years in three of the most populous states (Victoria (VIC), New South Wales (NSW) and Queensland) [23,52–54]. In this study, we identified higher rates of *S*. *sonnei* in males than females, particularly in those aged 30 to 64 years, which is the species responsible for several of the recent outbreaks among MSM in Australian communities. The eastern jurisdictions of Australia have a higher estimated proportion of adult gay men in the population compared to the western states and territories, with gay men tending to form more highly populated geospatial communities in urban areas compared to rural and regional areas [55]. It is vital to control the spread of shigellosis amongst MSM due to the increasing occurrence of drug resistant strains such as extensively drug-resistant *S*. *sonnei*, resistant to all recommended oral antibiotics, which has been reported among MSM in NSW and VIC [56,57].

The availability of species level information on shigellosis cases was strongly influenced by changes in diagnostic testing methodologies over time. Since 2014, there was an increase in cases of untyped *Shigella*, coinciding with increased use of culture independent testing methods for shigellosis diagnosis. Several studies have demonstrated the increased capability of CIDT in identifying the aetiology of diarrhoeal patients when compared to culture dependent methods, with estimates ranging from two to four times when using CIDT [58–60]. This may have contributed to the overall increase in cases that was observed since the widespread use of CIDT from 2014. CIDT are easy to perform, fast, sensitive and reliable, which makes them a valuable diagnostic tool. However, they are generally only able to identify the causative agent of an infection, without providing more granular level information such as pathogen strain, serotype or genotype, which is important to understand disease trends, detect outbreaks and monitor antimicrobial resistance patterns [61]. In addition, CIDT are unable to differentiate between *Shigella* and EIEC, which are closely related and genetically constitute the same species. Whole genome sequencing (WGS) allows public health agencies to obtain detailed epidemiological data for surveillance and investigation. WGS-based characterisation of *Shigella* species and serotypes provides higher-resolution and more accurate data than conventional biochemical and serological testing methods. The process to obtain this information is faster as WGS does not require the application of multiple subtyping methodologies [62]. Additionally, WGS can differentiate between *Shigella* and EIEC, which have many biochemical properties and virulence genes in common [62,63]. It is important to note however that WGS can only be applied on cultured isolates, which highlights the importance of continuing culture until metagenomics approaches become more available.

Differences between states and territories in the reporting of data presents some limitations for this study, particularly in making accurate comparisons between jurisdictions. For instance, the laboratory diagnostic method field in the NNDSS was applied inconsistently between jurisdictions, with some of these applications potentially resulting in an underestimation of cases diagnosed using CIDT and others resulting in an overestimate of cases. As the data used in this study is collected for surveillance purposes, detailed information on the protocols used by each laboratory for the detection of *Shigella* spp. is not available. However, in Australia, only a limited number of nucleic acid tests for faecal pathogens are approved for use in diagnostic laboratories [64]. Further, in order for nucleic acid tests (including those for *Shigella* spp.) to be used in Australian laboratories, the tests generally require accreditation from a national accreditation authority for each laboratory undertaking testing. This ensures compliance with international best practice and means that only high-quality tests are used in Australian laboratories.

Inconsistent interpretation of the surveillance case definition by some states presents another limitation of this study and may have particularly impacted the trends observed in the latter years of this study. Throughout the majority of the study period, the surveillance case definition for a ‘confirmed’ case of shigellosis was ‘*isolation or detection of Shigella species*’. The broad nature of this wording led a few jurisdictions to transmit cases that were detected using nucleic acid testing only to NNDSS, which, prior to the inclusion of a ‘probable’ category in the case definition in 2018, was recorded by the system as a ‘confirmed’ case. This primarily occurred from late 2017 until the implementation of the new case definition in mid-2018. Therefore, the observed increase in shigellosis cases between 2017 and mid-2018 may be in part attributable to the inadvertent classification of cases that were diagnosed using nucleic acid testing only as a ‘confirmed’ case of shigellosis. The differing testing practices between jurisdictions is evident when looking at the prevalence of untyped *Shigella*. For instance, VIC, NT and Tasmania all had a pronounced increase in the proportion of untyped *Shigella* from 2014 onwards, which is attributable to the uptake of PCR testing in these jurisdictions. The differential use of CIDT in jurisdictions, yielding a higher proportion of unknown *Shigella* species, will also impact the observed notification ratio of known species, particularly the two most prevalent species, S. *sonnei* and S. *flexneri*. For instance, in 2014, the use of CIDT in NSW was less than that in NT, which means that the contribution of the NT to notifications of S. *flexneri* is reduced compared to NSW due to a higher number of notifications of untyped *Shigella* in the NT.

Another limitation of the study was the lack of clinical information on cases. The nature of the data used in this study (i.e. data collected for public health surveillance purposes rather than for individual patient level management), meant that clinical information was not available for analysis, thereby limiting our ability to comment on clinical characteristics, such as the presence of symptoms, treatments, disease progression and severity.

Returning travellers are a high risk group for shigellosis [23]. To examine this trend on a national level, data on the place of acquisition of shigellosis cases was collected for this study. However the lack of completeness of this field limited its reliability for analysis and meaningful interpretation.

## Conclusion

Since 2001, notification rates of shigellosis have increased in Australia. This study has contributed to improving the understanding of the characteristics of shigellosis activity, including identifying high risk groups such as young children and First Nations Australians. Higher rates of transmission were also observed in males than females for *S*. *sonnei*, potentially reflecting transmission among MSM, which has been widely reported in Australia. The study observed a considerable increase in untyped *Shigella* in recent years which coincided with an increased use of CIDT. This highlights the implications of culture independent testing on shigellosis surveillance, with CIDT leading to a reduction in the availability of species level information. Enhancements in surveillance and laboratory practices, such as through the application of the revised surveillance case definition and use of WGS, will provide further insights into the epidemiological features of shigellosis in Australia, thereby enabling the implementation of targeted and efficient prevention and control interventions.

## Supporting information

S1 Fig Notification rate of shigellosis per 100,000 population, by sex and jurisdiction, Australia, 2001–2019. (DOCX)

S2 Fig Crude notification rates (dots) and negative binomial regression margins plots (lines with 95% CI) of *S*. *flexneri*, Australia 2001–2019. (DOCX)

S3 Fig Predicted notification rates of shigellosis per 100,000 population with 95% CI, by 5 year age group and species, Australia, 2001–2019. (DOCX)

S4 Fig Notification rate of shigellosis per 100,000 population, by jurisdiction, Australia, 2001–2019. (DOCX)

S5 Fig State and territory crude (dots) and predicted (lines with 95% CI) notification rates per 100,000 population, Australia, 2001–2019. (DOCX)

S6 Fig Proportion of shigellosis notifications, by jurisdiction and species, Australia, 2001–2019. (DOCX)

S1 Table Notification rate ratios estimated using negative binomial regression of *S*. *flexneri* by gender, age, state and time, 2001–2019. (DOCX)
