# Supplementary figures and images for: The changing epidemiology of shigellosis in Australia, 2001–2019
Source: PLoS Negl Trop Dis. 2023 Mar 1;17(3):e0010450. doi: 10.1371/journal.pntd.0010450 (PMC10010521; doi:10.1371/journal.pntd.0010450)

S1 Fig. Notification rate of shigellosis per 100,000 population, by sex and jurisdiction, Australia, 2001-2019


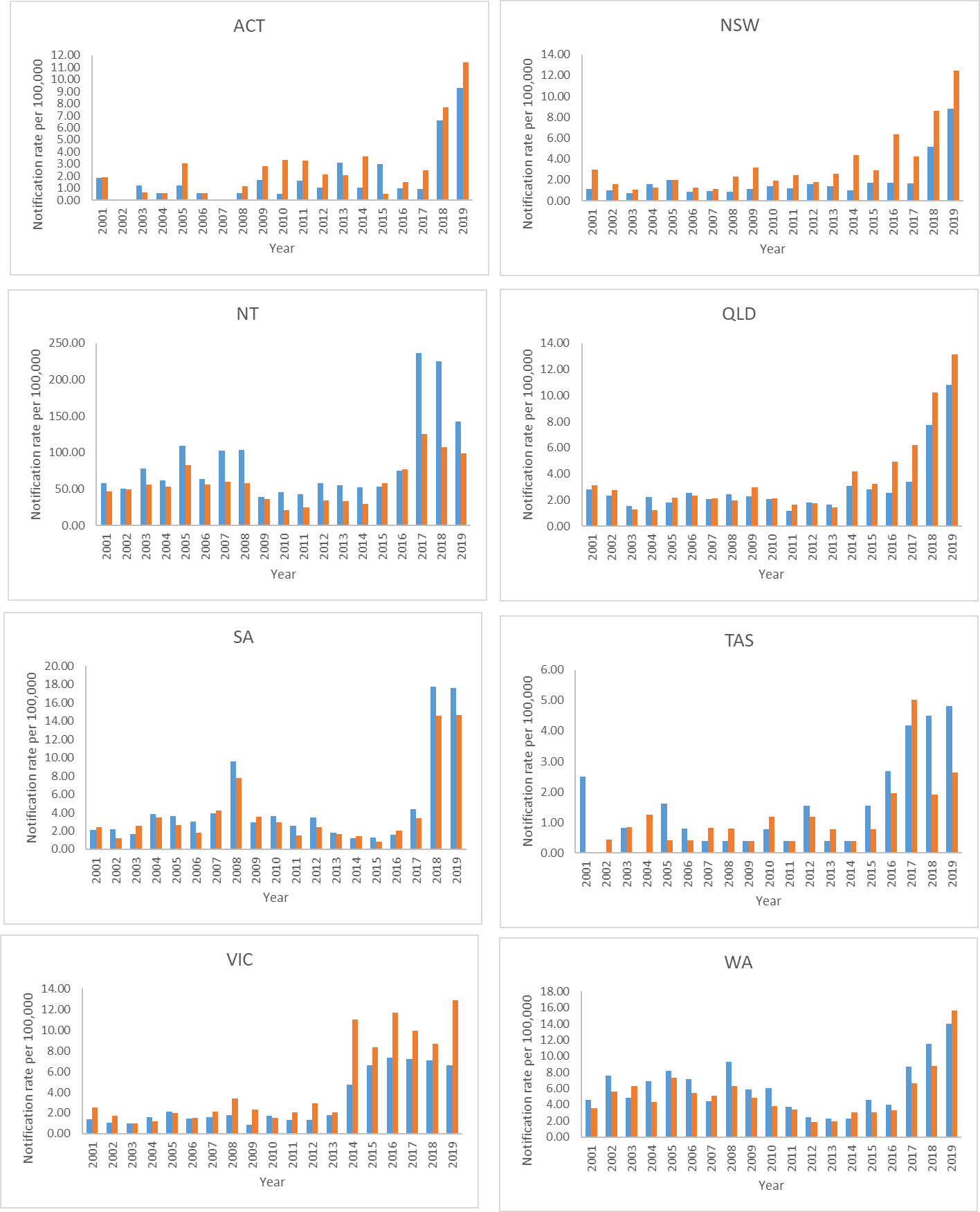


##
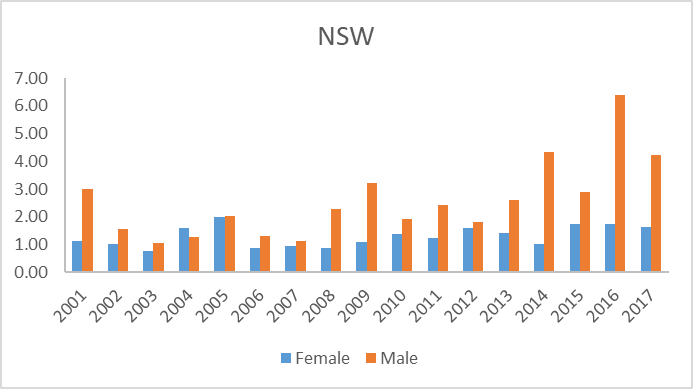

Supplement: S1 Fig — (DOCX) [file pntd.0010450.s001.docx]

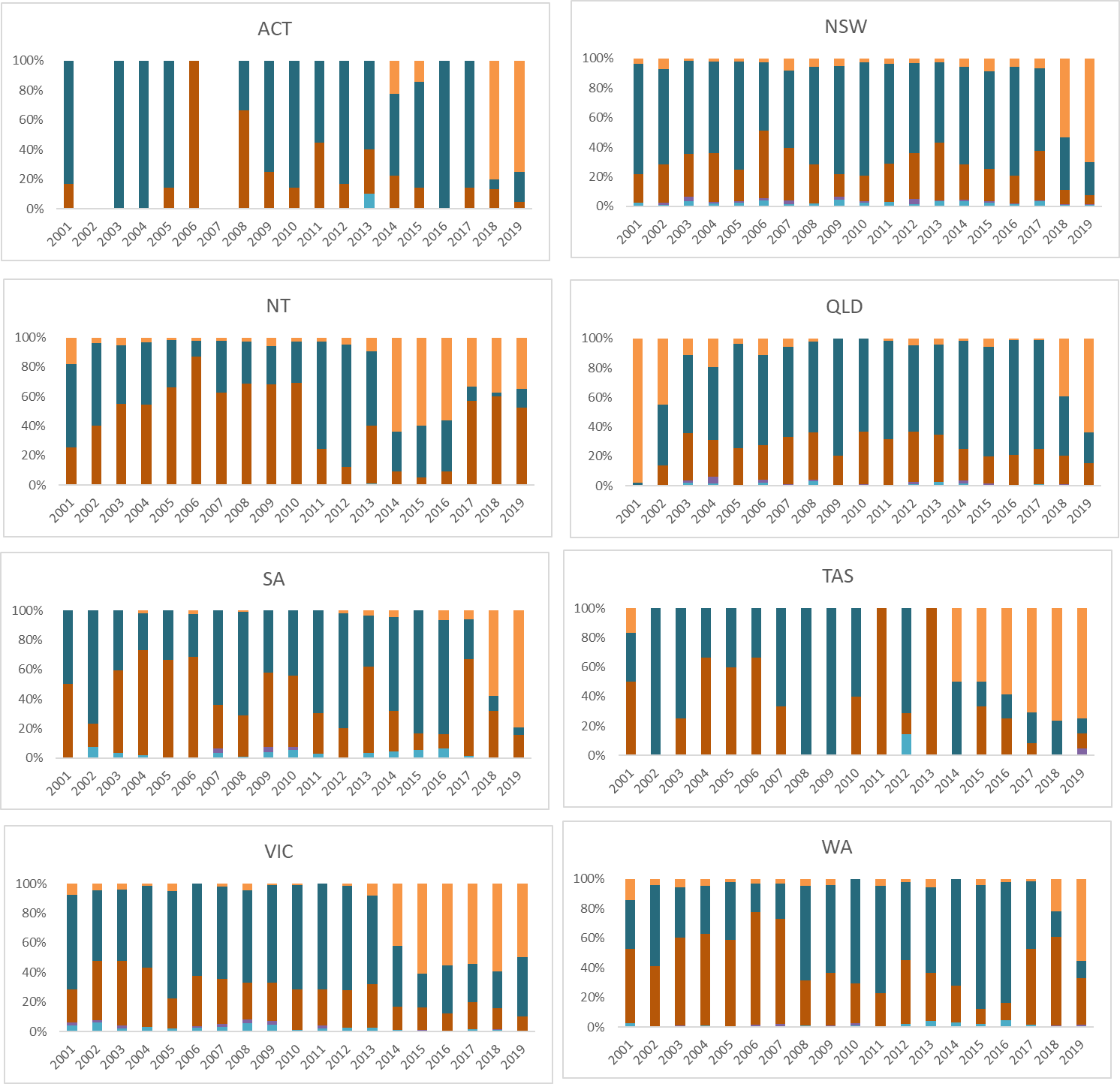
**S6 Fig. Proportion of shigellosis notifications, by jurisdiction and species, Australia, 2001-2019**


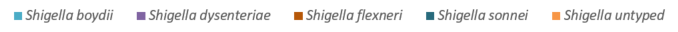

Supplement: S6 Fig — (DOCX) [file pntd.0010450.s006.docx]
