# Supplementary material for: The changing epidemiology of shigellosis in Australia, 2001–2019
Source: PLoS Negl Trop Dis. 2023 Mar 1;17(3):e0010450. doi: 10.1371/journal.pntd.0010450 (PMC10010521; doi:10.1371/journal.pntd.0010450)
Supplement: S2 Fig — (DOCX) [file pntd.0010450.s002.docx]

**S2 Fig. Crude notification rates (dots) and negative binomial regression margins plots (lines with 95% CI) of *S. flexneri*, Australia 2001-2019**

**
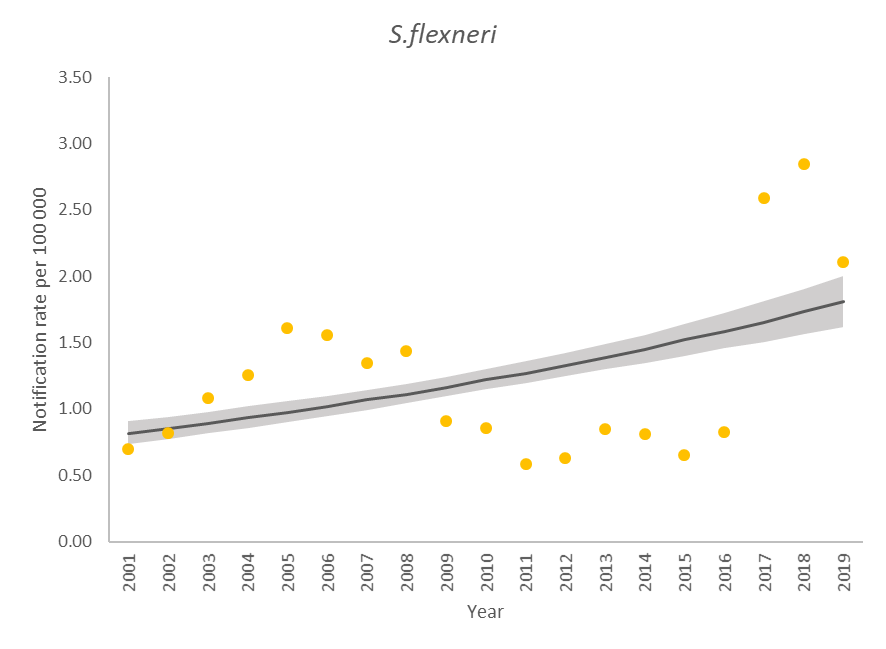
**
