# Supplementary material for: The changing epidemiology of shigellosis in Australia, 2001–2019
Source: PLoS Negl Trop Dis. 2023 Mar 1;17(3):e0010450. doi: 10.1371/journal.pntd.0010450 (PMC10010521; doi:10.1371/journal.pntd.0010450)

**S3 Fig. Predicted notification rates of shigellosis per 100,000 population with 95% CI, by 5 year age group and species, Australia, 2001-2019.** *(Note the differing scale of the y-axis for S. boydii and S. dysenteriae, compared to S. sonnei and S. flexneri).*


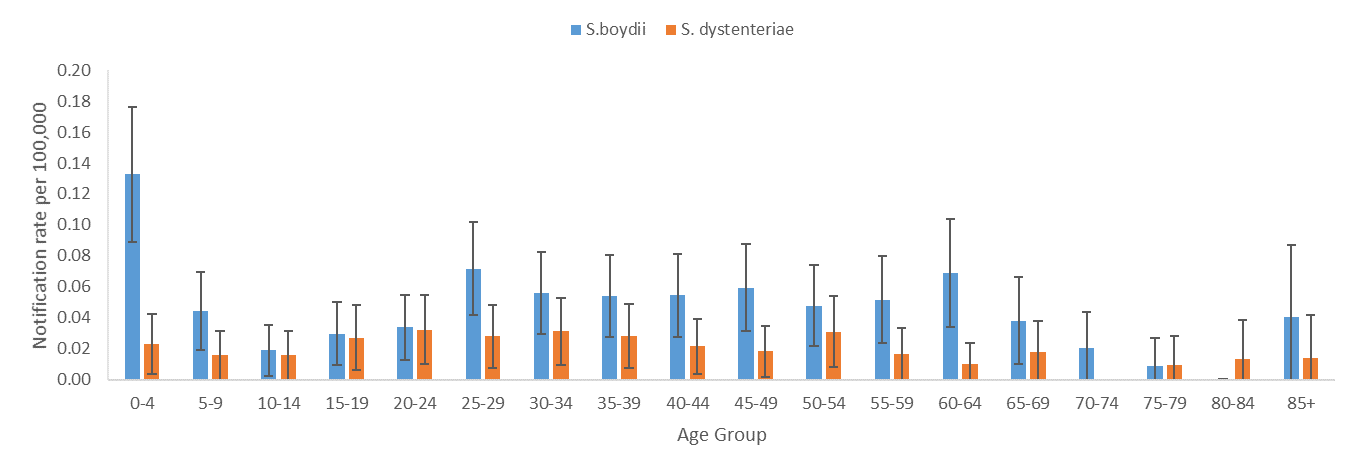

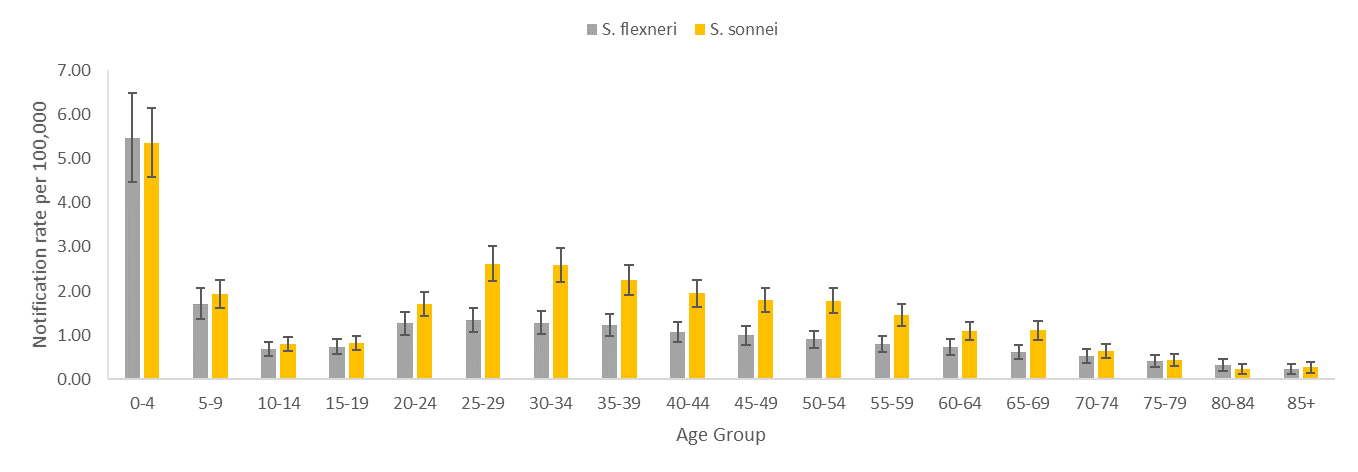

Supplement: S3 Fig — (DOCX) [file pntd.0010450.s003.docx]
