# Supplementary material for: The changing epidemiology of shigellosis in Australia, 2001–2019
Source: PLoS Negl Trop Dis. 2023 Mar 1;17(3):e0010450. doi: 10.1371/journal.pntd.0010450 (PMC10010521; doi:10.1371/journal.pntd.0010450)
Supplement: S4 Fig — (DOCX) [file pntd.0010450.s004.docx]

**S4 Fig. Notification rate of shigellosis per 100,000 population, by jurisdiction, Australia, 2001-2019^1^**

**
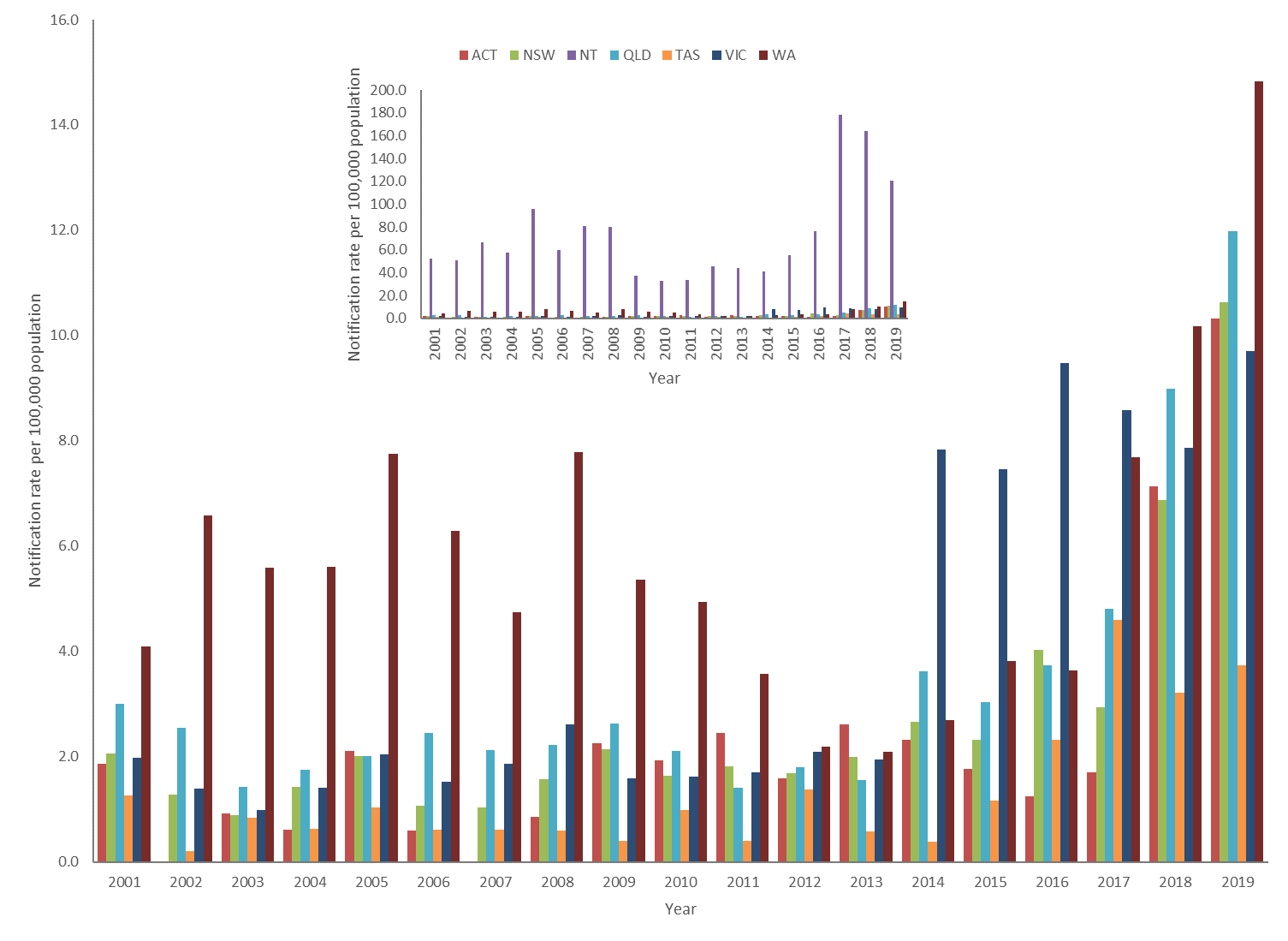
**

^1^ Due to considerably higher rates, Northern Territory (NT) is excluded in the main graph to allow better visualization of the rates in the other jurisdictions. NT is included in the inset graph to show a comparison between all jurisdictions.
