# Supplementary material for: The changing epidemiology of shigellosis in Australia, 2001–2019
Source: PLoS Negl Trop Dis. 2023 Mar 1;17(3):e0010450. doi: 10.1371/journal.pntd.0010450 (PMC10010521; doi:10.1371/journal.pntd.0010450)

S5 Fig. State and territory crude (dots) and predicted (lines with 95% CI) notification rates per 100,000 population, Australia, 2001-2019

***S.flexneri***

***
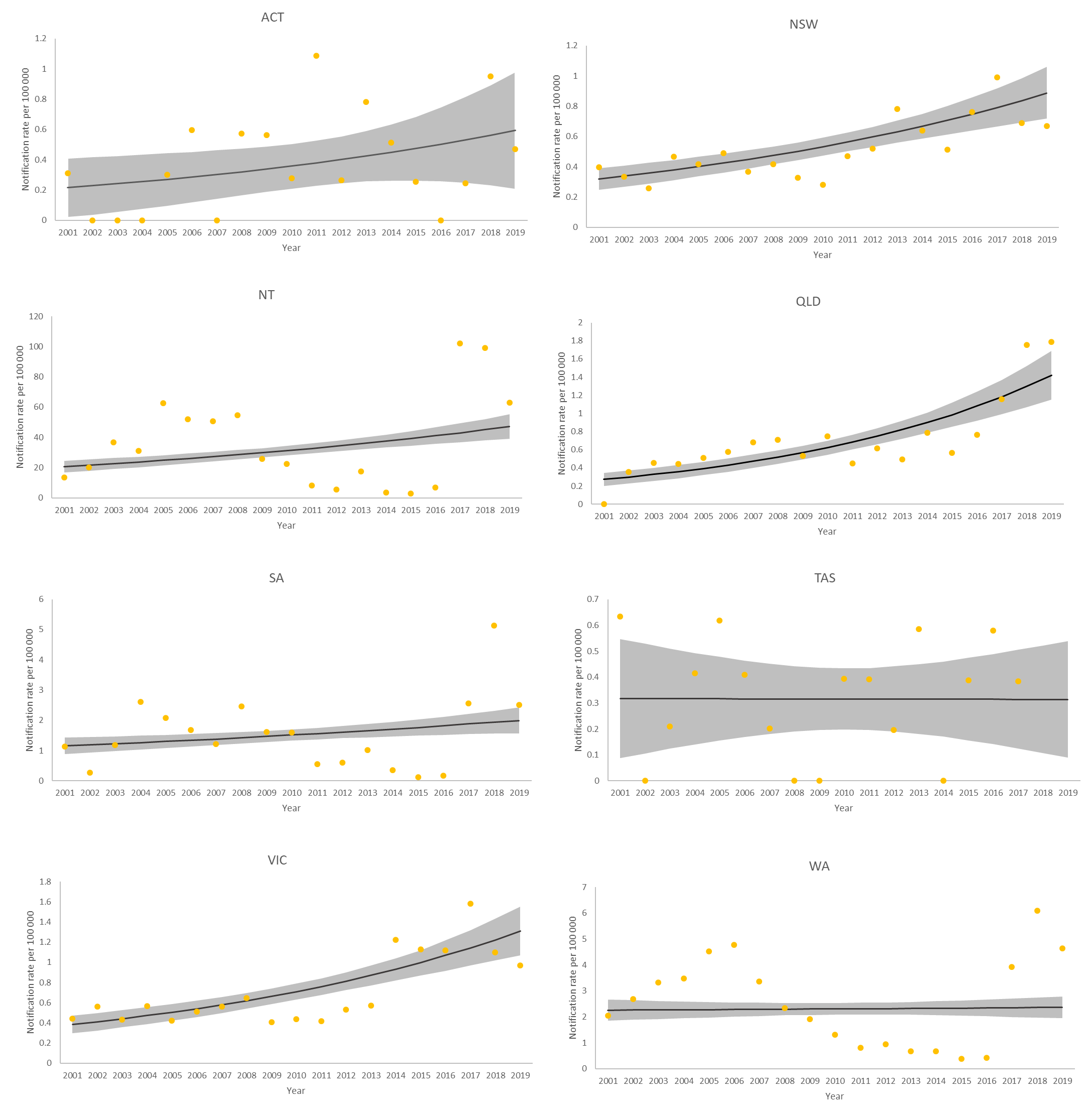
***

***S.sonnei***

**
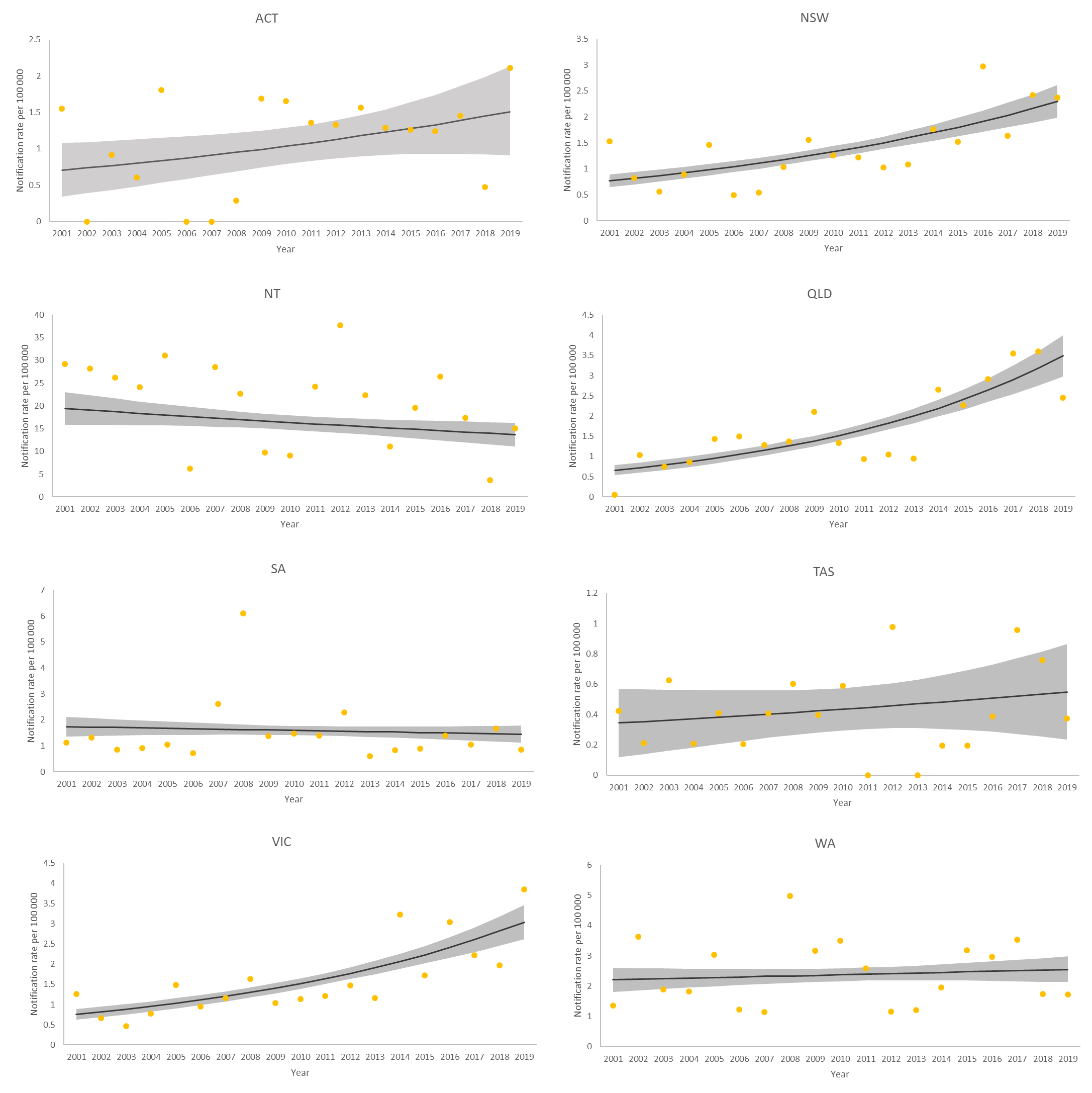
**

***S.dysenteriae***

##
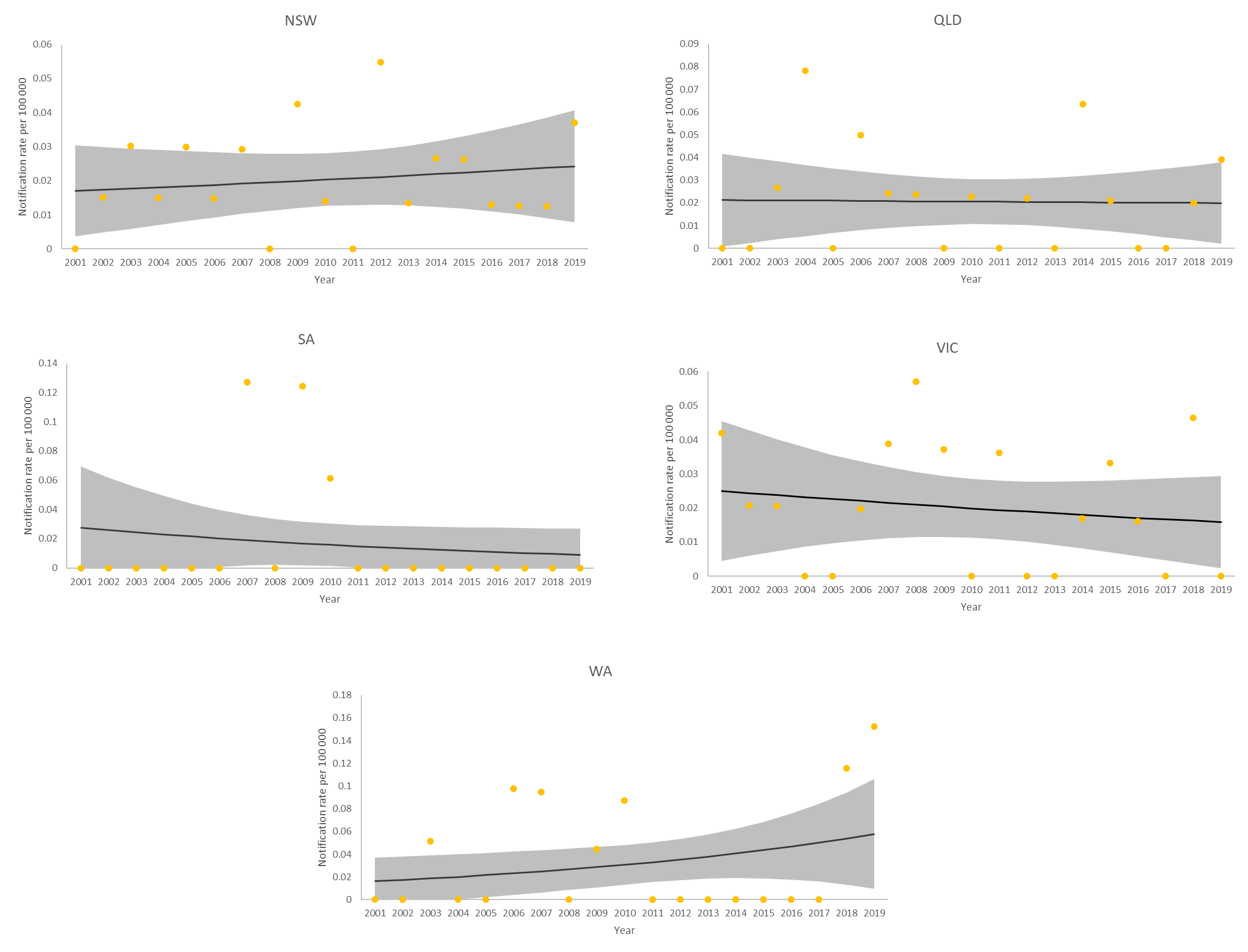


***S.boydii***


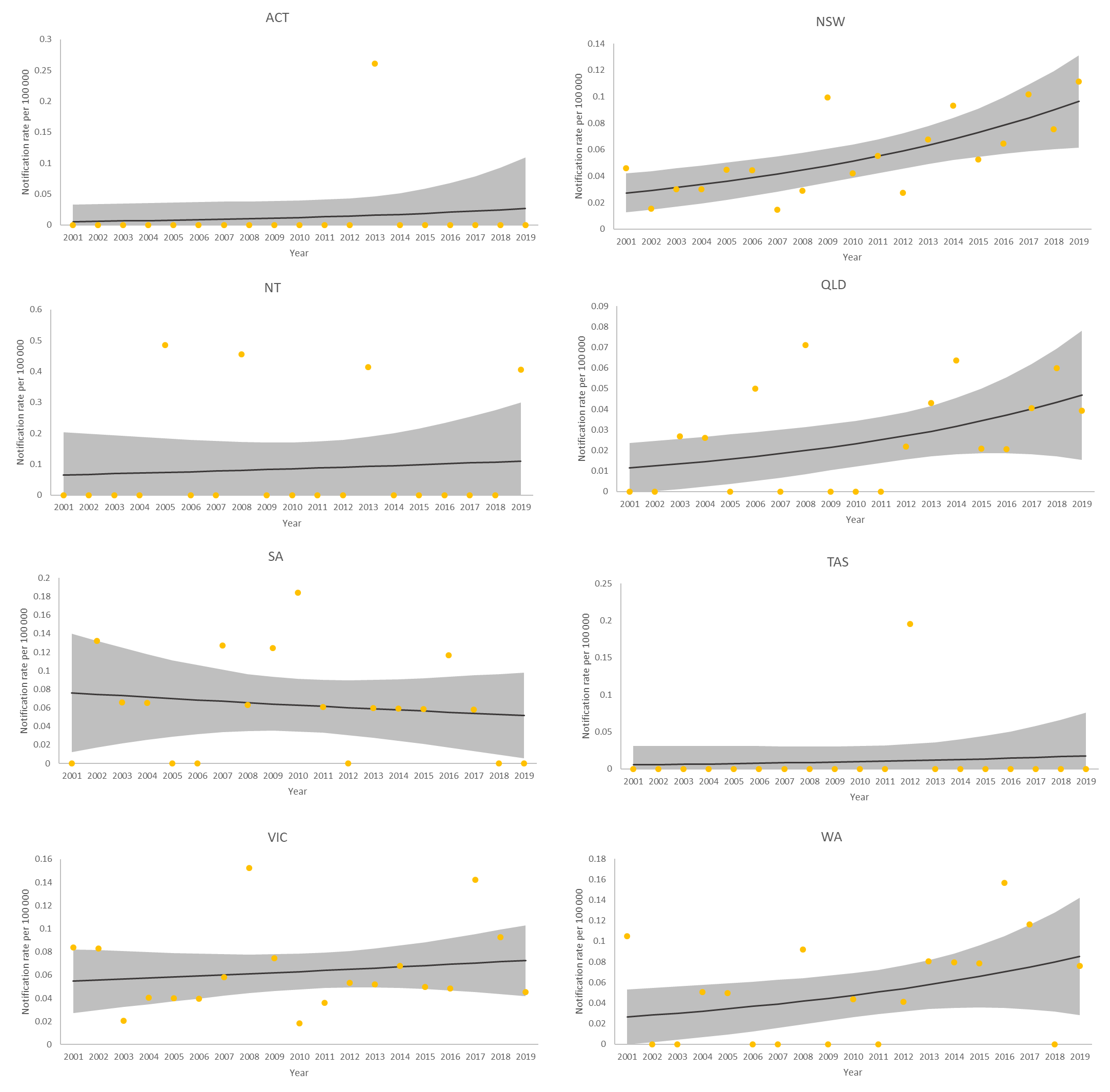

Supplement: S5 Fig — (DOCX) [file pntd.0010450.s005.docx]
