# Supplementary material for: The changing epidemiology of shigellosis in Australia, 2001–2019
Source: PLoS Negl Trop Dis. 2023 Mar 1;17(3):e0010450. doi: 10.1371/journal.pntd.0010450 (PMC10010521; doi:10.1371/journal.pntd.0010450)
Supplement: S1 Table — (DOCX) [file pntd.0010450.s007.docx]

**S1 Table. Notification rate ratios estimated using negative binomial regression of *S. flexneri* by gender, age, state and time, 2001-2019**

|  | ***S.flexneri*** |
| --- | --- |
|  | **IRR (95% CI)** |
| **Age groups (reference 0-4)** | |
| 5-9 | 0.36 (0.30-0.44) |
| 10-14 | 0.15 (0.12-0.19) |
| 15-19 | 0.17 (0.14-0.21) |
| 20-24 | 0.28 (0.23-0.35) |
| 25-29 | 0.30 (0.25-0.37) |
| 30-34 | 0.29 (0.24-0.36) |
| 35-39 | 0.28 (0.22-0.34) |
| 40-44 | 0.24 (0.20-0.30) |
| 45-49 | 0.23 (0.18-0.28) |
| 50-54 | 0.21 (0.17-0.25) |
| 55-59 | 0.19 (0.15-0.23) |
| 60-64 | 0.18 (0.14-0.23) |
| 65-69 | 0.16 (0.12-0.20) |
| 70-74 | 0.14 (0.11-0.19) |
| 75-79 | 0.12 (0.08-0.16) |
| 80-84 | 0.09 (0.06-0.14) |
| 85+ | 0.07 (0.04-0.11) |
| **Jurisdiction (reference=NSW)** | |
| ACT | 0.67 (0.27-1.71) |
| NT | 64.87 (48.39-86.96) |
| QLD | 0.86 (0.61-1.21) |
| SA | 3.61 (2.60-5.00) |
| TAS | 0.99 (0.47-2.12) |
| VIC | 1.20 (0.87-1.66) |
| WA | 7.07 (5.30-9.44) |
| **Trend over time by state and territory (2001-2019)** | |
| ACT | 1.06 (0.98-1.14) |
| NSW | 1.06 (1.04-1.08) |
| NT | 1.05 (1.03-1.06) |
| QLD | 1.10 (1.07-1.12) |
| SA | 1.03 (1.01-1.05) |
| TAS | 1.00 (0.93-1.07) |
| VIC | 1.07 (1.05-1.09) |
| WA | 1.00 (0.99-1.02) |
| **Sex (reference=female)** | |
| Male | 1.03 (0.95-1.13) |
